# Layer-Matched A2 Shade Compatibility Across 3Y/4Y/5Y Multilayer Zirconia: CIEDE2000 Color Differences Correlated with Y_2_O_3_ Content (EDS), Phase Constitution (XRD), and Grain Size (FE-SEM)

**DOI:** 10.3390/dj14040226

**Published:** 2026-04-10

**Authors:** Carlos Roberto Luna-Dominguez, Suria Sarahi Oliver-Rivas, Omaika Victoria Criollo-Barrios, Gerardo Alberto Salvador Gomez-Lara, Ricardo de Jesús Figueroa-Lopez, Jorge Humberto Luna-Dominguez

**Affiliations:** Faculty of Dentistry, Autonomous University of Tamaulipas, Av. Universidad Esq. con Blvd, Adolfo Lopez Mateos S/N, Tampico 89337, Tamaulipas, Mexico; cldominguez@docentes.uat.edu.mx (C.R.L.-D.); suria.oliver@uat.edu.mx (S.S.O.-R.); omaika.criollo@uat.edu.mx (O.V.C.-B.); gerardo.gomez@docentes.uat.edu.mx (G.A.S.G.-L.); ricardo.figueroa@uat.edu.mx (R.d.J.F.-L.)

**Keywords:** zirconia, CAD/CAM, yttrium content, color match, tooth shade, multilayer zirconia

## Abstract

**Objective:** This in vitro study aimed to compare the layer-matched color compatibility of three 3Y/4Y/5Y multilayer zirconia grades marketed in shade A2. **Materials and Methods:** Disc specimens (18 mm × 1.5 mm) were milled from pre-shaded multilayer zirconia blanks (Katana™ Multi-Layered Zirconia; Kuraray Noritake Dental Inc., Tokyo, Japan) in three grades: UTML (5Y), STML (4Y), and HTML (3Y). Twelve discs per grade were polished and measured on a neutral-gray background (Munsell N7) using a dental spectrophotometer (VITA Easyshade Advance 4.0; VITA Zahnfabrik, Bad Säckingen, Germany) at the incisal, middle, and cervical thirds. Color differences were calculated using CIEDE2000 (ΔE_00_). Yttria content (wt%) was determined using EDS (JSM-7800F; JEOL Ltd., Tokyo, Japan), and phases were assessed using XRD (X’Pert PRO; Malvern Panalytical, Almelo, The Netherlands); microstructure and grain size were examined using FE-SEM after thermal etching. **Statistics:** A two-way mixed-design ANOVA with Bonferroni adjustment (α = 0.05) was conducted. **Results:** A significant incisal-to-cervical gradient was observed within each grade (*p* < 0.001), whereas layer-matched inter-material differences were small (all ΔE00 < 1.0), i.e., below the commonly accepted perceptibility threshold. EDS confirmed the expected stepwise decrease in Y_2_O_3_ from UTML to HTML, accompanied by corresponding changes in phase constitution and grain size. **Conclusions:** Despite compositional and microstructural differences, the three multilayer zirconia grades showed no clinically perceptible layer-matched color differences, supporting their combined use in extended rehabilitations while maintaining the natural-like color gradient across the multilayer blank.

## 1. Introduction

The adoption of fully digital workflows and the broad availability of metal-free CAD/CAM restorative materials have reshaped contemporary prosthetic dentistry by enabling conservative preparations, streamlined fabrication, and improved standardization of restorative outcomes [1,2]. Among these materials, monolithic zirconia is widely used for single crowns and fixed dental prostheses (FDPs) due to its high flexural strength, favorable fracture behavior, reduced risk of veneer chipping compared with veneered systems, and compatibility with digital manufacturing chains [3,4].

Zirconium dioxide (zirconia) is a polymorphic ceramic that exhibits monoclinic (m), tetragonal (t), and cubic (c) phases depending on temperature; stabilization of the high-temperature phases at room temperature is achieved using oxide dopants such as yttrium oxide (Y_2_O_3_) [5,6,7,8]. In dental zirconia, increasing yttria content is commonly used to define material grades (e.g., 3Y, 4Y, and 5Y), which are associated with distinct phase assemblages and microstructures that directly influence optical and mechanical behavior [9,10,11]. Higher Y_2_O_3_ contents generally increase the contribution of cubic-stabilized zirconia and may promote grain growth after sintering, which can enhance translucency by reducing light scattering at grain boundaries; however, this typically occurs at the expense of transformation toughening and may reduce fracture resistance and strength [12,13,14,15]. Conversely, 3Y zirconia is predominantly tetragonal and benefits more from stress-induced transformation toughening, usually exhibiting higher mechanical performance but lower translucency than higher-yttria grades, while 4Y zirconia represents an intermediate balance [10,11,13,15].

Multilayer zirconia blanks have been developed to mimic the incisal-to-cervical optical stratification of natural teeth by incorporating a vertical gradient in color and translucency within the blank [16,17,18,19,20]. In the Katana™ multilayer system (Kuraray Noritake, Tokyo, Japan), three related products are available with different nominal yttria contents: Ultra-Translucent Multilayered (UTML, ≈5 mol% Y_2_O_3_; “5Y”), Super-Translucent Multilayered (STML, ≈4 mol% Y_2_O_3_; “4Y”), and High-Translucent Multilayered (HTML, ≈3 mol% Y_2_O_3_; “3Y”) zirconia [6,9]. These grades are recommended for different clinical indications, ranging from highly esthetic anterior restorations (UTML) to restorations requiring higher strength, particularly in posterior or longer-span scenarios (HTML), with STML positioned between them [6]. Although the multilayer concept is intended to promote esthetic integration across the restoration height, clinicians may also wish to combine different grades within the same rehabilitation (e.g., using higher-translucency zirconia in esthetic regions and higher-strength zirconia in load-bearing areas). Under these circumstances, even subtle differences in phase composition and microstructure between grades may translate into measurable and potentially perceptible color discrepancies when adjacent components are fabricated from different zirconia grades, despite being marketed in matching classical shades (e.g., A2).

Instrumental color assessment in dentistry is commonly performed using the CIELAB color space (L*, a*, and b*), and color differences are frequently expressed using the CIEDE2000 metric (ΔE_00_), which better aligns with visual perception than earlier formulas [21]. For dental applications, ΔE_00_ perceptibility and acceptability thresholds of approximately 0.8 and 1.8, respectively, have been proposed as 50:50% limits under controlled observation conditions [21,22,23,24]. However, quantitative evidence remains limited regarding (1) cross-grade color compatibility among UTML, STML, and HTML within the same multilayer system and nominal shade and (2) layer-resolved incisal-to-cervical gradients within each grade, particularly when interpreted alongside compositional and microstructural characteristics.

Therefore, the aim of this in vitro study was to evaluate color similarity among three multilayered zirconia materials (UTML (5Y), STML (4Y), and HTML (3Y)) manufactured in the same nominal shade A2, both between materials and across the incisal, middle, and cervical regions within each material. In addition, differences in yttrium content, crystalline phase distribution, and grain morphology were examined to provide compositional and microstructural context for any observed color differences. The null hypothesis was that no clinically relevant color differences according to established CIEDE2000 perceptibility and acceptability thresholds would be detected, either among the three zirconia grades or among the regions within each material.

## 2. Materials and Methods

This experimental in vitro comparative study was conducted on three multilayer zirconia grades manufactured in the same nominal shade A2 (UTML, STML, and HTML). For the colorimetric component, a 3 × 3 mixed factorial design with repeated measures was used, with zirconia grade as the between-specimen factor and measurement region within the same disc (incisal, middle, and cervical) as the within-specimen factor. Measurements were performed in the initial condition (post-sintering and polishing) without artificial aging protocols. The sintered disc was the experimental and primary analytical unit; repeated readings within each region were averaged to improve repeatability without inflating the effective sample size (pseudoreplication control). An a priori power analysis for the planned grade × region interaction in a mixed-design ANOVA (G*Power 3.1 (Heinrich-Heine University Düsseldorf, Düsseldorf, Germany)) was performed using α = 0.05, power (1 − β) = 0.80, effect size f = 0.25, three groups, three repeated measurements, correlation among repeated measures ρ = 0.50, and ε = 1.0, yielding a minimum required sample of N = 36 (n = 12 per grade) for the color measurements. In total, 63 sintered discs were fabricated (21 per grade): 36 discs were allocated to color measurements (n = 12 per grade), and an independent set of 27 discs (n = 9 per grade) was allocated to characterization (XRD, FE-SEM/EDS, and grain size; n = 3 per grade per technique).

### 2.1. Materials

Three multilayer zirconia grades from the same product family and nominal shade (A2) were evaluated: Ultra-Translucent Multilayered (UTML, 5Y), Super-Translucent Multilayered (STML, 4Y), and High-Translucent Multilayered (HTML, 3Y) zirconia (Katana™ multilayered zirconia system; Kuraray Noritake Dental Inc., Tokyo, Japan). Manufacturer-declared yttria content, flexural strength ranges, and recommended clinical indications for each grade are summarized in Table 1.

### 2.2. Specimen Preparation

**Figure 1:**
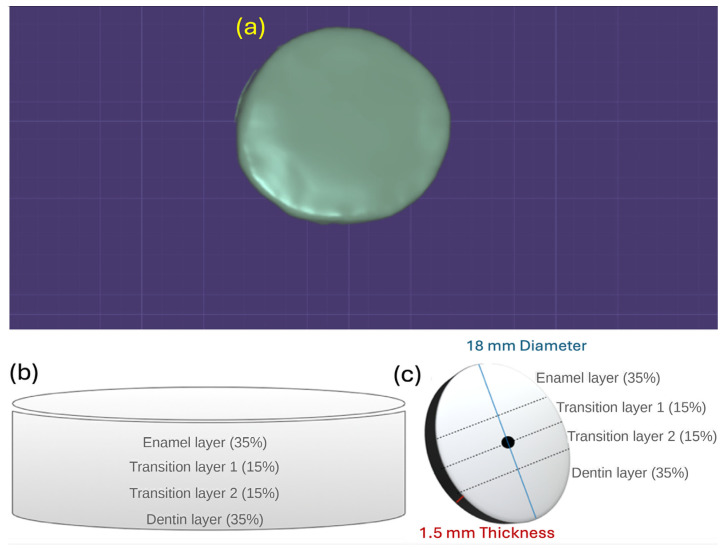
CAD model and multilayer architecture of zirconia disc specimens. (**a**) Digital rendering of the 18 mm × 1.5 mm disc generated in DentalCAD 3.0 Galway (exocad GmbH, Darmstadt, Germany). (**b**) Schematic cross-section illustrating the nominal distribution of the enamel layer (35%), transition layer 1 (15%), transition layer 2 (15%), and dentin layer (35%) within the multilayer blank. (**c**) Orientation of the layered structure in the finished specimen, showing the position of the measurement axis across the disc diameter.

A circular disc-shaped specimen with target sintered dimensions of 18.0 mm in diameter and 1.50 mm in thickness was designed using DentalCAD 3.0 Galway (exocad GmbH, Darmstadt, Germany) and used as the master file for all experimental groups (Figure 1a). For each zirconia grade, twelve discs were fabricated (n = 12 per material; total n = 36) from A2, 98 mm × 18 mm pre-sintered blanks. Milling was performed using a five-axis unit (CORiTEC 350i, imes-icore GmbH, Eiterfeld, Germany).

To preserve the multilayer architecture, the CAD design was oriented so that the incisal surface of the disc corresponded to the incisal region of the blank (Figure 1b,c). Accordingly, the nominal layered distribution (enamel layer 35%, transition layer 1 15%, transition layer 2 15%, and dentin layer 35%) was maintained along the measurement axis across the disc.

Sintering schedules were grade-specific and followed the manufacturer’s recommendations (Table 2). All firings were carried out in the same high-temperature furnace (TT-1800; Nabertherm GmbH, Lilienthal, Germany) calibrated to ±2 °C across the working chamber. Total cycle time comprised heating at 10 °C·min^−1^, followed by dwell time at the specified peak temperature and natural cooling to <200 °C before removal.

After sintering, discs were separated from the blanks using a low-speed handpiece (~10,000 rpm) with a fine-grit diamond bur under continuous water irrigation to avoid thermal damage. To minimize surface-related optical variation, all specimens were standardized to a polished surface using a two-step diamond-impregnated rubber polishing system (EVE Diapol, EVE Ernst Vetter GmbH, Pforzheim, Germany) at ~10,000 rpm and light hand pressure: 30 s with the coarse (green) polisher followed by 30 s with the fine (red) polisher. The specimens were rotated 90° between steps to promote uniform surface treatment.

Final thickness was verified using a high-precision digital micrometer (Mitutoyo Corporation, Kawasaki, Kanagawa, Japan; resolution 0.001 mm) at three points (center, incisal third, and cervical third). Only specimens within ±0.05 mm of the nominal thickness (1.50 mm) were included. Accepted discs were ultrasonically cleaned in distilled water (40 kHz, 120 W; Elmasonic S 40 R, Elma GmbH, Singen, Germany) for 10 min, air-dried, and stored in a dust-free enclosure prior to measurements and further analyses.

### 2.3. Color Measurement

Color was quantified using a chairside spectrophotometer (VITA Easyshade Advance 4.0; VITA Zahnfabrik, Bad Säckingen, Germany) operating in CIELAB mode under the instrument’s standard “Tooth” measurement geometry with D65-equivalent illumination. Before each measurement series, the device was calibrated against its factory white reference according to the manufacturer’s instructions. To minimize background influence (show-through), each specimen was placed on a neutral gray card (Munsell N7). All measurements were performed in a temperature- and humidity-controlled room (23 ± 1 °C; 50 ± 5% relative humidity) by a single trained operator. The order of specimen and region measurements was randomized.

Each disc was analyzed at three predetermined vertical locations corresponding to the incisal (upper third), middle (body), and cervical (lower third) regions. At each location, the probe was held perpendicular and flush to the polished surface with consistent light pressure; the built-in positioning ring ensured a constant measuring aperture (Ø 5 mm). Three consecutive readings were obtained per location, and their arithmetic mean represented the region’s L*, a*, and b* coordinates. In the CIELAB color space, L* denotes lightness (0 = black, 100 = white), a* the red–green axis (+a* = red, −a* = green), and b* the yellow–blue axis (+b* = yellow, −b* = blue).

Color differences (ΔE00) were calculated using the following CIEDE2000 formula with weighting factors $k_{L}=k_{C}=k_{H}=1$ :

(1) $${∆E}_{00}\sqrt{{\left(\frac{{∆L}^{\prime }}{k_{L}S_{L}}\right)}^{2}+\;{\left(\frac{{∆C}^{\prime }}{k_{C}S_{C}}\right)}^{2}+\;{\left(\frac{{∆H}^{\prime }}{k_{H}S_{H}}\right)}^{2}+RT\left(\frac{{∆C}^{\prime }}{k_{C}S_{C}}\right)\left(\frac{{∆H}^{\prime }}{k_{H}S_{H}}\right)}$$

where $\Delta L^{\prime }$ , $\Delta C^{\prime }$ , and $\Delta H^{\prime }$ are the differences in lightness, chroma, and hue (after CIEDE2000 corrections); $S_{L}$ , $S_{C}$ , and $S_{H}$ are the corresponding weighting functions; and $R_{T}$ is the rotation term accounting for the interaction between chroma and hue differences.

Two comparison strategies were applied: (1) intra-material comparisons among incisal, middle, and cervical regions within each zirconia grade to characterize the multilayer color gradient and (2) inter-material comparisons in corresponding regions (incisal-to-incisal, middle-to-middle, and cervical-to-cervical) among UTML, STML, and HTML to evaluate cross-grade shade compatibility within the same nominal shade (A2).

For clinical interpretation, previously reported CIEDE2000 thresholds for dental applications were adopted: ΔE_00_ ≈ 0.8 as the 50:50% perceptibility threshold (PT) and ΔE_00_ ≈ 1.8 as the 50:50% acceptability threshold (AT). Accordingly, ΔE_00_ < 0.8 was considered visually imperceptible, values between 0.8 and 1.8 were considered perceptible but clinically acceptable, and values ≥1.8 were considered potentially unacceptable.

### 2.4. Elemental and Phase Analysis

For each zirconia grade (UTML, STML, and HTML), characterization was performed on independent sets of sintered discs to minimize sample-preparation artifacts and cross-contamination between techniques. Specifically, three discs per grade (n = 3) were allocated for X-ray diffraction (XRD), and three additional discs per grade (n = 3) were designated for FE-SEM/EDS. All specimens were randomly selected from the same manufacturing batch.

### 2.5. X-Ray Diffraction (XRD)

Phase composition was assessed using a PANalytical X’Pert PRO diffractometer (Malvern Panalytical, Almelo, The Netherlands) with Cu Kα radiation (λ = 1.5406 Å) operated at 40 kV and 30 mA. Specimens were mounted flat on a zero-background holder and scanned over a 2θ range of 20–120° using a step size of 0.02° and a counting time of 3 s per step. To explore potential phase differences across the multilayer architecture, diffraction patterns were obtained from both the incisal and cervical surfaces of each disc. Phase identification was performed qualitatively by comparison with reference patterns, focusing on characteristic reflections of tetragonal zirconia (e.g., ${\left(101\right)}_{t}$ , ${\left(110\right)}_{t}$ , ${\left(112\right)}_{t}$ , ${\left(211\right)}_{t}$ ) and cubic-stabilized zirconia (e.g., ${\left(111\right)}_{c}$ , ${\left(200\right)}_{c}$ , ${\left(220\right)}_{c}$ ). The presence of monoclinic zirconia was assessed via inspection of characteristic m-ZrO_2_ reflections in the ~28–31° 2θ region.

### 2.6. FE-SEM/EDS

For elemental analysis, discs were sputter-coated with a thin (~10 nm) Au/Pd layer and mounted on aluminum stubs. Analyses were performed using a JSM-7800F field-emission scanning electron microscope (JEOL Ltd., Tokyo, Japan) equipped with an Oxford X-Max 80 mm^2^ silicon drift EDS detector. EDS measurements were obtained from the polished central region at 15 kV, a working distance of 8 mm, and a probe current of 10 nA. For each disc, three non-overlapping fields were analyzed (a total of nine measurements per grade), using a live time of 60 s per field. Spectra were processed using a standardless ZAF correction procedure; Au/Pd peaks from the coating were excluded from quantification. Elemental concentrations were reported as oxide weight percent (wt%) (ZrO_2_, Y_2_O_3_). The final composition for each grade was obtained by averaging the nine measurements.

### 2.7. Microstructural Analysis

Microstructural characterization was performed to examine grain morphology and quantify average grain size. Three additional sintered discs per zirconia grade (n = 3) were designated for this evaluation.

#### 2.7.1. Thermal Etching and Surface Preparation

To reveal grain boundaries, polished discs were thermally etched in a high-temperature box furnace by heating at 10 °C·min^−1^ to 1200 °C, holding for 1 h, and then cooling at 10 °C·min^−1^ to room temperature. After etching, specimens were sputter-coated with a thin (~10 nm) Au/Pd layer to minimize charging during FE-SEM imaging.

#### 2.7.2. SEM Imaging

Micrographs were acquired using a JSM-7800F field-emission scanning electron microscope (FE-SEM) (JEOL Ltd., Tokyo, Japan). Images were obtained in secondary-electron mode at a nominal magnification of 10,000×. The accelerating voltage and working distance were set according to the acquisition parameters used for the micrographs analyzed (reporting the exact values shown in the image, e.g., 2.0–3.0 kV and WD 6.4–6.8 mm for the representative images). For each disc, five non-overlapping fields of view were randomly selected from the polished central region, yielding 15 micrographs per zirconia grade (five fields per disc × three discs).

#### 2.7.3. Grain Size Determination

Average grain size ( $\bar{d}$ ) was quantified using the line-intercept (Heyn) method in accordance with ASTM E112. Each SEM micrograph (10,000×) was first calibrated in ImageJ 1.54r software (National Institutes of Health, Bethesda, MD, USA) using the embedded 1 µm scale bar so that all subsequent measurements were expressed in micrometers (µm). A region of interest (ROI) was defined by excluding the image annotation/scale-bar area and avoiding the outer borders to prevent edge-related artifacts.

A set of test lines was superimposed on each calibrated micrograph within the ROI (five horizontal and five vertical lines, resulting in a total = 10 lines). The total test line length (Lt, µm) was recorded as the sum of the lengths of all test lines. Grain-boundary intersections (N) were counted manually as the number of times a test line crossed a clearly discernible grain boundary. Intersections occurring exactly at line endpoints were not counted to avoid double-counting, and tangential contacts where a test line ran along a boundary were not counted unless a clear crossing was observed.

The mean linear intercept length ( $\bar{l}$ ) was calculated as follows:

(2) $$\bar{l}=\mathrm{Lt}/N$$

Assuming an approximately isotropic polycrystalline microstructure with predominantly equiaxed grains, the mean grain diameter was derived using the following stereological relationship:

(3) $$\bar{d}=(\pi /2)\cdot \bar{l}\approx 1.57\cdot \bar{l}$$

Quantitative analysis was performed on 15 micrographs per zirconia grade (five non-overlapping fields per disc × three discs), ensuring >300 grain-boundary intercepts per grade to obtain robust grain size estimates. Grain size data were summarized as mean ± standard deviation across micrographs. In addition, qualitative microstructural features (e.g., equiaxed vs. faceted morphology, heterogeneous grain size distribution, residual porosity, and inclusions) were recorded.

### 2.8. Statistical Analysis

Statistical analyses were performed at a significance level of α = 0.05 using IBM SPSS Statistics, version 25 (IBM Corp., Armonk, NY, USA). Data are reported as mean ± standard deviation (SD) unless otherwise stated. For color coordinates, the experimental unit was the disc specimen (n = 12 per zirconia grade). For each specimen, L*, a*, and b* values were obtained at three repeated locations (upper/incisal, middle/body, and lower/cervical thirds), with each location value defined as the arithmetic mean of three consecutive spectrophotometer readings.

The effects of zirconia grade (UTML, STML, and HTML; between-subject factor) and region (upper, middle, and lower; within-subject repeated factor), as well as their interaction (grade × region), on L*, a*, and b* coordinates were evaluated using a two-way mixed-design ANOVA. Assumptions were assessed by inspection of residuals for approximate normality (e.g., Q–Q plots and/or Shapiro–Wilk test) and by testing homogeneity of variance for the between-subject factor (e.g., Levene’s test). For the within-subject factor, sphericity was assessed (Mauchly’s test), and, if violated, the Greenhouse–Geisser correction was applied. When statistically significant main effects were identified, pairwise comparisons were conducted with Bonferroni adjustment for multiple testing.

Color differences were computed as CIEDE2000 (ΔE_00_) using Equation (1) with parametric weighting factors kL = kC = kH = 1. In the present study, ΔE_00_ values were calculated from the mean CIELAB coordinates for each material and region to summarize perceptual distances between (1) regions within a material (intra-material gradient) and (2) materials within the same region (inter-material compatibility). Therefore, ΔE_00_ outcomes were primarily interpreted descriptively relative to established clinical thresholds (PT ≈ 0.8; AT ≈ 1.8) rather than subjected to inferential hypothesis testing.

For grain size, the statistical unit was the micrograph (15 micrographs per zirconia grade; five non-overlapping fields per disc × three discs). Mean grain size values were compared among zirconia grades using Welch’s one-way ANOVA to account for potential heteroscedasticity. When the omnibus test was significant, heteroscedasticity-robust post hoc pairwise comparisons (e.g., Games–Howell) were performed. Mean differences were reported together with 95% confidence intervals (CIs) where applicable.

## 3. Results

### 3.1. CIELAB Coordinates (L*, a*, b*)

**Figure 2:**
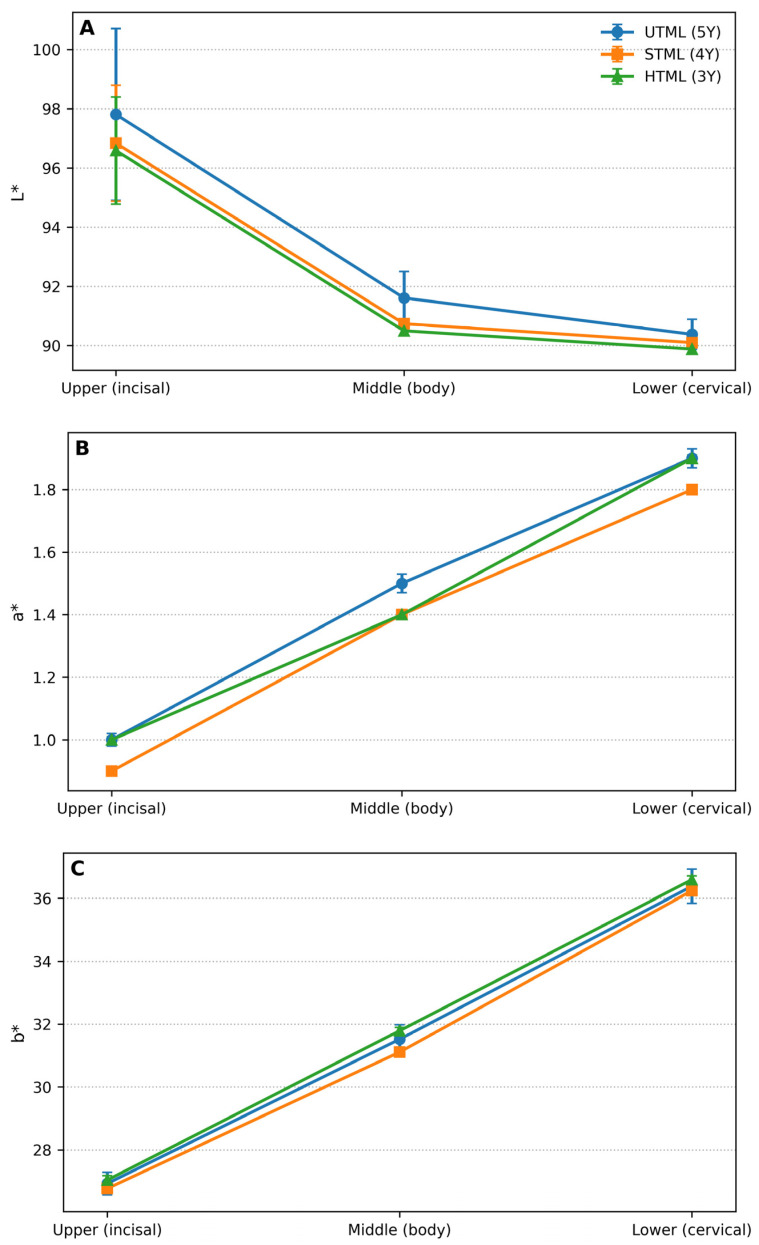
Marginal mean profiles of (**A**) L*, (**B**) a*, and (**C**) b* coordinates across the upper (incisal), middle (body), and lower (cervical) thirds for UTML (5Y), STML (4Y), and HTML (3Y) zirconia (shade A2). Data are presented as mean ± SD (n = 12 per material).

The mean (±SD) CIELAB coordinates for UTML, STML, and HTML zirconia across the upper (incisal), middle (body), and lower (cervical) thirds are reported in Table 3 and illustrated in Figure 2. A mixed-model ANOVA identified a significant main effect of region for all three coordinates (L*, a*, and b*; all *p* < 0.001), confirming the presence of a consistent incisal-to-cervical color gradient within the multilayer discs.

#### 3.1.1. Lightness (L*)

L* values decreased from the upper third (incisal) to the middle and lower thirds in all zirconia grades (Table 3; Figure 2A). The main effect of region on L* was significant (F(2,66) = 265.25, *p* < 0.001). Bonferroni-adjusted pairwise comparisons showed significant differences among all regions (all *p* < 0.001), with an average decrease of 6.13 units from upper to middle and 6.96 units from upper to lower; the middle-to-lower decrease was 0.83 units.

A significant main effect of material was also observed for L* (F(2,33) = 5.23, *p* = 0.011). Post hoc comparisons indicated that UTML exhibited slightly higher L* than HTML (mean difference = 0.94, *p* = 0.012), whereas UTML–STML (*p* = 0.078) and STML–HTML (*p* = 1.000) differences were not statistically significant. The region × material interaction was not significant (F(4,66) = 0.30, *p* = 0.88), indicating that the regional lightness gradient was comparable among the three zirconia grades.

#### 3.1.2. Red–Green Coordinate (a*)

The a* coordinate increased progressively toward the cervical third for all materials (Table 3; Figure 2B). Region significantly influenced a* (F(2,66) = 18,546.42, *p* < 0.001). All region pairwise comparisons were significant (all *p* < 0.001), corresponding to average increases of 0.47 units from upper to middle, 0.90 units from upper to lower, and 0.43 units from middle to lower.

Material also had a significant effect on a* (F(2,33) = 668.00, *p* < 0.001). Bonferroni-adjusted comparisons showed that UTML presented higher a* values than STML (mean difference = 0.10, *p* < 0.001) and HTML (mean difference = 0.03, *p*< 0.001), while HTML presented higher a* values than STML (mean difference = 0.07, *p* < 0.001). The region × material interaction was significant (F(4,66) = 32.65, *p* < 0.001), reflecting small differences in the magnitude of the a* regional increase among zirconia grades; however, the direction of change (increasing a* from upper to lower thirds) remained consistent across materials.

#### 3.1.3. Yellow–Blue Coordinate (b*)

b* values increased markedly from the upper to the lower third in all groups (Table 3; Figure 2C). The main effect of region was significant (F(2,66) = 21,318.34, *p* < 0.001). Bonferroni-adjusted comparisons confirmed significant differences among all regions (all *p* < 0.001), with mean increases of 4.56 units from upper to middle, 9.49 units from upper to lower, and 4.93 units from middle to lower.

A significant main effect of material was detected for b* (F(2,33) = 10.33, *p* < 0.001). Post hoc comparisons indicated higher b* for HTML than for STML (mean difference = 0.44, *p* < 0.001), whereas UTML did not differ significantly from STML (*p* = 0.055) or HTML (*p* = 0.144). The region × material interaction was significant (F(4,66) = 4.10, *p* = 0.005), indicating minor differences in the b* regional profiles among zirconia grades.

Overall, the upper/incisal third exhibited the highest lightness (higher L*) and the lowest chromatic coordinates (lower a* and b*), while the lower/cervical third showed the lowest L* and the highest a* and b* values across all materials, confirming a pronounced built-in multilayer optical gradient within UTML, STML, and HTML specimens.

### 3.2. CIEDE2000 Color Differences (ΔE_00_)

**Figure 3:**
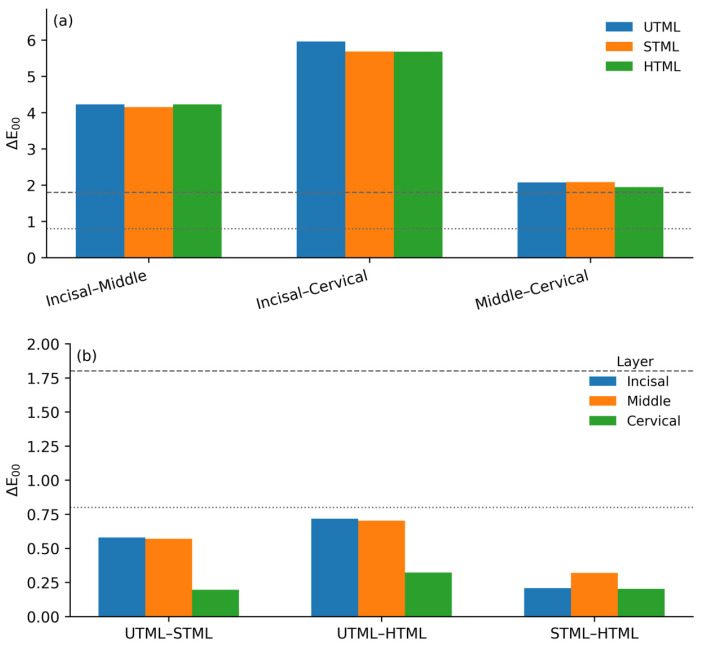
CIEDE2000 color differences (ΔE_00_) for multilayered zirconia. (**a**) Intra-material ΔE_00_ between incisal–middle, incisal–cervical, and middle–cervical thirds for UTML, STML, and HTML specimens. (**b**) Inter-material ΔE_00_ (UTML–STML, UTML–HTML, and STML–HTML) calculated separately for the incisal, middle, and cervical thirds. The dotted and dashed horizontal lines indicate the 50:50% perceptibility (ΔE_00_ = 0.8) and acceptability (ΔE_00_ = 1.8) thresholds, respectively.

Color differences (ΔE_00_) were calculated using the CIEDE2000 formula (Equation (1); $k_{L}=k_{C}=k_{H}=1$ ) based on the mean CIELAB coordinates of each material and layer to quantify the perceptual magnitude of the intra-material chromatic gradient across layers and the inter-material shade compatibility at equivalent layers (Table 4; Figure 3).

#### 3.2.1. Intra-Material Color Differences Across Layers

All three zirconia grades exhibited pronounced intra-material color differences between layers (Figure 3a). The largest contrast consistently occurred between the incisal and cervical thirds, with ΔE_00_ values of 5.96 (UTML), 5.68 (STML), and 5.68 (HTML). Incisal–middle comparisons also showed high color differences (4.23, 4.16, and 4.23, respectively). Although smaller in magnitude, middle–cervical differences remained clearly detectable (2.08 for UTML, 2.09 for STML, and 1.95 for HTML). Notably, all intra-material layer contrasts exceeded the acceptability threshold (AT ≈ 1.8), confirming that the multilayer blanks produced a clinically evident polychromatic gradient from the incisal to cervical regions.

#### 3.2.2. Inter-Material Color Differences Within Equivalent Layers

In contrast, inter-material color differences within the same layer were minimal (Figure 3b). Across all pairwise material comparisons (UTML–STML, UTML–HTML, and STML–HTML), ΔE_00_ values ranged from 0.20 to 0.72, remaining below the perceptibility threshold (PT ≈ 0.8). The largest differences were observed for UTML–HTML in the incisal (ΔE_00_ = 0.72) and middle (ΔE00 = 0.70) thirds, whereas cervical-layer differences were uniformly low (≤0.32). These results indicate that, despite statistically significant differences in individual CIELAB coordinates (Section 3.1), the three zirconia grades displayed visually imperceptible color discrepancies when compared at corresponding layers in the same nominal shade (A2).

### 3.3. Yttrium Oxide Content (EDS)

**Figure 4:**
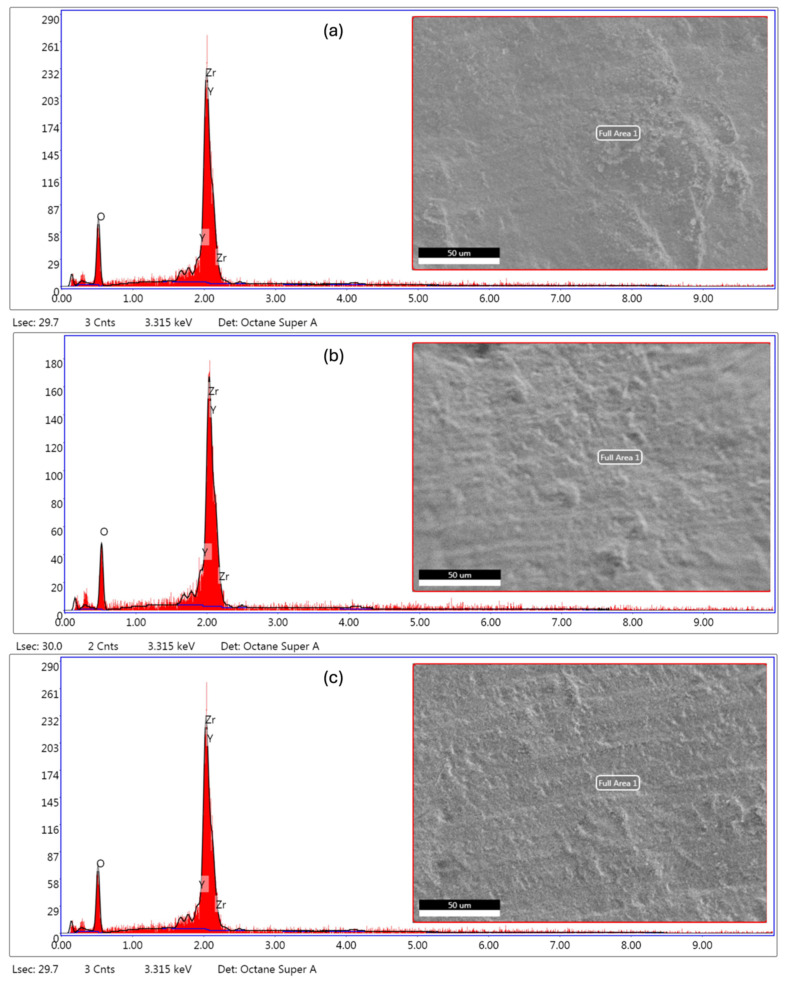
Representative FE-SEM micrographs of the analyzed areas (**right**) and corresponding EDS spectra (**left**) for (**a**) UTML (5Y), (**b**) STML (4Y), and (**c**) HTML (3Y) multilayer zirconia. Spectra show the expected signals for O and Zr with detectable Y contributions. Scale bar = 50 µm.

Representative EDS spectra confirmed the expected elemental constituents of yttria-stabilized zirconia, with dominant signals corresponding to O, Zr, and Y in all groups and no apparent peaks attributable to extraneous contaminants (Figure 4). Quantitative EDS, expressed as oxide weight percent (wt%), revealed a clear stepwise decrease in yttrium oxide content across the three zirconia grades (Table 5). When disc-level averages (each obtained from three non-overlapping EDS fields) were pooled (n = 3 discs per grade), UTML showed the highest Y_2_O_3_ fraction (12.21 ± 0.09 wt%), followed by STML (10.04 ± 0.13 wt%) and HTML (7.07 ± 0.11 wt%). The corresponding ZrO_2_ fractions increased complementarily (87.79 ± 0.09 wt%, 89.96 ± 0.13 wt%, and 92.93 ± 0.11 wt%, respectively). Overall, the low within-group dispersion (SD ≤ 0.13 wt%) indicates good compositional consistency within each grade and confirms the intended stabilizer gradient of the multilayer zirconia system (UTML > STML > HTML).

### 3.4. Phase Composition (XRD)

**Figure 5:**
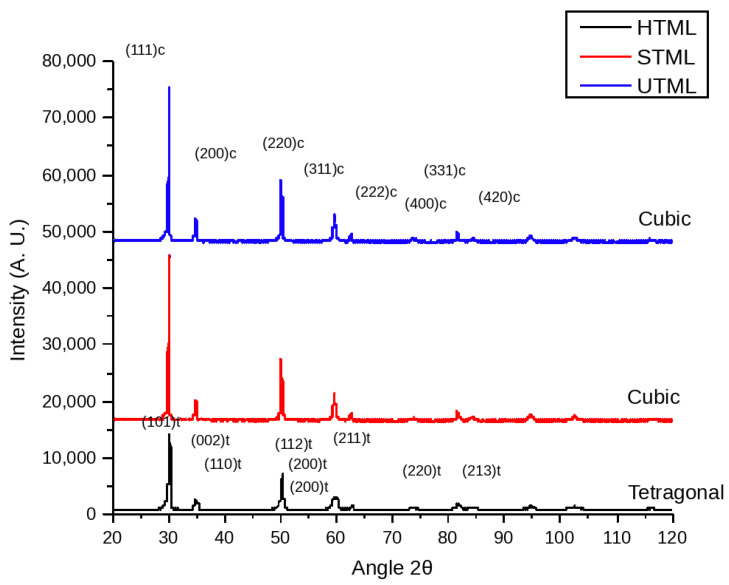
X-ray diffraction (XRD) patterns of UTML, STML, and HTML zirconia after sintering. Peaks indexed to cubic (c) and tetragonal (t) zirconia are indicated. Diffractograms are vertically offset for clarity.

Representative X-ray diffraction (XRD) patterns of the three multilayer zirconia grades are shown in Figure 5. All diffractograms were consistent with yttria-stabilized zirconia, with reflections attributable to tetragonal (t) and/or cubic (c) zirconia. Importantly, no monoclinic ZrO_2_ reflections were detected in the characteristic low-angle region (≈28–31° 2θ), indicating that the specimens retained the stabilized high-temperature phases after sintering and did not exhibit detectable transformation to the monoclinic phase under the conditions evaluated.

Clear differences in phase assemblage were observed among the three grades. UTML (5Y) exhibited a diffraction pattern dominated by cubic-phase reflections, including intense peaks indexed as (111)c, (200)c, (220)c, and (311)c, together with additional higher-angle cubic reflections (e.g., (222)c, (400)c, (331)c, and (420)c). In contrast, HTML (3Y) showed a pattern characteristic of predominantly tetragonal zirconia, with prominent tetragonal reflections such as (101)t, (002)t/(110)t, (112)t, and (211)t, and comparatively minimal evidence of cubic stabilization. STML (4Y) displayed an intermediate behavior, with coexisting tetragonal reflections and discernible cubic contributions, consistent with its intermediate stabilizer level. Overall, the qualitative phase hierarchy observed via XRD indicated the following: higher cubic contribution in UTML, intermediate in STML, and predominantly tetragonal in HTML. This result agrees with the measured yttria contents (UTML > STML > HTML) and supports the expected stabilizer-dependent shift from transformation-toughenable tetragonal microstructures toward cubic-stabilized zirconia.

### 3.5. Microstructural Analysis and Grain Size Determination

#### 3.5.1. FE-SEM Microstructural Observations

**Figure 6:**
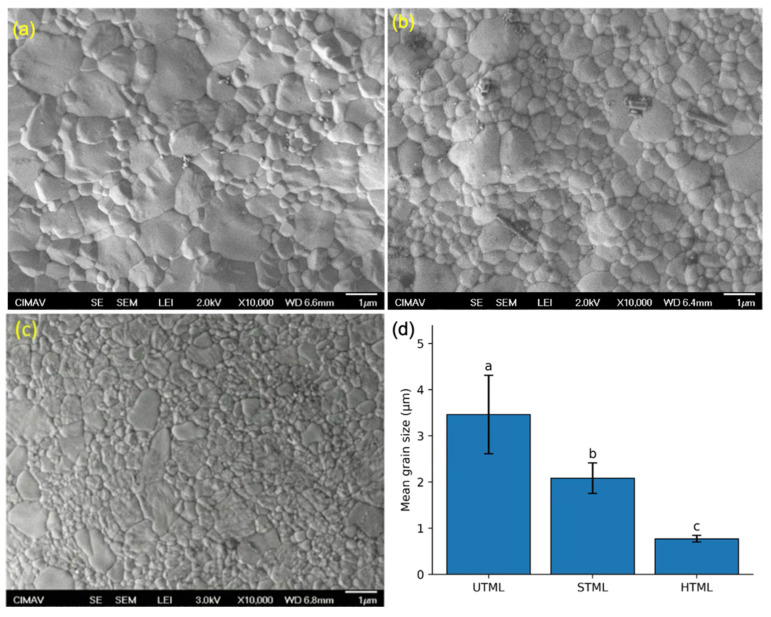
Representative FE-SEM micrographs of thermally etched zirconia surfaces (10,000×). (**a**) UTML (5Y), (**b**) STML (4Y), and (**c**) HTML (3Y). Scale bar = 1 µm. (**d**) Mean grain size (µm) of UTML, STML, and HTML zirconia determined using the line-intercept method. Bars represent mean ± SD. Different letters indicate statistically significant differences between zirconia grades (Welch ANOVA, *p* < 0.001; post hoc pairwise comparisons, *p* < 0.001).

Representative FE-SEM micrographs of the thermally etched zirconia surfaces are shown in Figure 6. All materials exhibited a dense polycrystalline microstructure with clearly delineated grain boundaries at 10,000× magnification, and no apparent open porosity was observed in the analyzed fields. Distinct differences in grain morphology and distribution were evident among zirconia grades. UTML (5Y) displayed the coarsest microstructure, with large faceted grains interspersed with smaller grains, consistent with a broad grain size distribution (Figure 6a). STML (4Y) showed an intermediate microstructure characterized by moderately sized, predominantly equiaxed grains with less pronounced size heterogeneity (Figure 6b). In contrast, HTML (3Y) exhibited the finest and most homogeneous microstructure, with closely packed small grains and a comparatively narrow grain size distribution (Figure 6c).

#### 3.5.2. Grain Size (Line-Intercept Method)

Quantitative grain size measurements corroborated the qualitative FE-SEM observations (Figure 6d). Mean grain size (±SD) values were 3.46 ± 0.85 µm for UTML, 2.08 ± 0.33 µm for STML, and 0.77 ± 0.07 µm for HTML. Welch’s ANOVA revealed a significant effect of zirconia grade on grain size (F(2, 19.57) = 176.56, *p* < 0.001). Post hoc pairwise comparisons indicated that UTML exhibited significantly larger grains than STML (mean difference = 1.38 µm, 95% CI: 0.91–1.85, *p* < 0.001) and HTML (mean difference = 2.69 µm, 95% CI: 2.22–3.16, *p* < 0.001). In addition, STML showed significantly larger grains than HTML (mean difference = 1.31 µm, 95% CI: 0.84–1.78, *p* < 0.001). Overall, the standard deviation was highest for UTML, indicating greater within-group grain size variability, whereas HTML showed the lowest dispersion, consistent with a narrower grain size distribution.

## 4. Discussion

This study evaluated whether three multilayer zirconia grades from the same commercial system and nominal shade A2 (UTML/5Y, STML/4Y, and HTML/3Y) exhibit a clinically meaningful incisal-to-cervical chromatic gradient and layer-matched cross-grade color compatibility. Two outcomes are central. First, all grades demonstrated a pronounced internal gradient: intra-material ΔE_00_ values were 5.68–5.96 for incisal–cervical and 1.95–2.09 for middle–cervical comparisons, exceeding the commonly used acceptability threshold (AT ≈ 1.8) and therefore representing a clearly perceptible polychromatic transition within each disc. Second, inter-material differences at equivalent layers were consistently small (ΔE_00_ = 0.20–0.72), remaining below the perceptibility threshold (PT ≈ 0.8), supporting high cross-grade shade compatibility under the present measurement conditions. These findings collectively indicate that the null hypothesis should be rejected for the within-material (layer) contrasts but retained for the between-material (layer-matched) comparisons when interpreted using PT/AT criteria.

The magnitude of the intra-material ΔE_00_ gradients is explained by the consistent, directionally coherent CIELAB trends across all zirconia grades. From incisal (upper) to cervical (lower) thirds, L* decreased markedly (≈6.7–7.4 units), while a* and b* increased (≈+0.90 and ≈+9.4–9.6 units, respectively). This combined shift toward lower lightness and higher chroma cervically produces a strong perceptual transition that matches the design intent of multilayer blanks: a more enamel-like incisal zone and a more dentin-like cervical zone. Importantly, because these intra-material differences represent an intended gradient rather than a “mismatch,” ΔE_00_ values above AT in this context should be interpreted as evidence that the manufacturer’s polychromatic architecture is optically effective. Clinically, such built-in gradients may reduce reliance on external characterization (stains/glazes) to generate cervical chroma and incisal brightness; however, finishing procedures can still influence surface-dependent optical outcomes, so standardized polishing or glazing remains important when shade predictability is required [25,26,27].

It is also important to clarify the relationship between the nominal A2 shade designation and the CIELAB coordinates reported in this study. The A2 label (VITA Classical system) is provided by the manufacturer for the multilayer blanks, whereas there is no single fixed set of L*, a*, and b* values that uniquely defines A2 across devices and experimental conditions. CIELAB coordinates depend on measurement geometry/illumination, specimen thickness, surface finish, and background. Thus, values such as L* = 97.81, a* = 1.00, and b* = 26.93 should be interpreted as the measured coordinates of the incisal region of an A2-labeled zirconia disc under the standardized conditions used here (polished 1.50 mm discs on a neutral gray background, measured with VITA Easyshade Advance 4.0). For this reason, the clinical interpretation of the present work is based primarily on relative layer-matched ΔE_00_ differences between grades and regions. Future studies could directly quantify the absolute A2 match by measuring a VITA A2 shade tab (or certified reference) under identical conditions.

In contrast to the strong within-disc gradient, the layer-matched inter-material ΔE_00_ values were uniformly below PT, with the largest differences occurring between UTML and HTML in the incisal (ΔE00 = 0.72) and middle (ΔE00 = 0.70) thirds, which is still within the range expected to be visually imperceptible. This helps reconcile a key observation: although mixed-model ANOVA detected statistically significant effects of material on individual CIELAB coordinates (especially for a* and b*), these coordinate shifts were small in absolute magnitude and did not translate into clinically detectable color differences when expressed using the perceptually weighted CIEDE2000 metric. In other words, statistical significance under controlled in vitro conditions does not necessarily imply clinical relevance, particularly when variability is low, and sample sizes are sufficient to detect subtle differences. From a restorative perspective, the present layer-matched comparisons support the practical feasibility of mixing grades within the same rehabilitation (e.g., selecting a higher-translucency grade in esthetic zones and a more strength-oriented grade in load-bearing zones) without introducing visible shade discontinuities if thickness, surface finishing, and the layer alignment within the multilayer blank are maintained [28,29,30].

The added compositional and microstructural findings strengthen the mechanistic basis for interpreting why cross-grade shade compatibility can be preserved despite clear differences in stabilizer content and phase constitution. EDS confirmed a stepwise decrease in Y_2_O_3_ from UTML (12.21 ± 0.09 wt%) to STML (10.04 ± 0.13 wt%) and HTML (7.07 ± 0.11 wt%), which is consistent with the intended 5Y→4Y→3Y hierarchy. Correspondingly, XRD qualitatively demonstrated an increased cubic contribution in UTML, an intermediate tetragonal/cubic pattern in STML, and a predominantly tetragonal pattern in HTML, with no detectable monoclinic reflections after sintering. Notably, the new microstructural dataset (Section 3.5) showed that grain size followed the same hierarchy and differed significantly among grades: UTML presented the largest mean grain size (3.46 ± 0.85 µm), followed by STML (2.08 ± 0.33 µm), while HTML displayed the finest grains (0.77 ± 0.07 µm), with all pairwise comparisons significant (*p* < 0.001). The larger dispersion in UTML (SD 0.85 µm) also indicates a broader grain size distribution, which is consistent with the qualitative FE-SEM appearance of mixed large and smaller grains. These outcomes are consistent with the established stabilizer-dependent evolution of zirconia microstructure after sintering, where higher yttria contents tend to increase the fraction of cubic-stabilized zirconia and may promote grain growth [19,20]. Because cubic zirconia is optically isotropic (reduced birefringence relative to tetragonal zirconia) and grain size/grain boundaries influence light scattering, differences in yttria-phase grain size are expected to modulate light transport (e.g., translucency) and may contribute to subtle shifts in reflectance-based coordinates such as L* [25,26]. However, the fact that layer-matched ΔE_00_ values remained below PT across grades suggests that, within this commercial system, the underlying shade formulation and the multilayer color design are sufficiently aligned across 3Y/4Y/5Y products such that phase/microstructure differences do not produce clinically perceptible hue/chroma offsets under standardized reflectance conditions [6,13,22,25].

Overall, the present pattern aligns with previous investigations showing that multilayer/high-translucency zirconias exhibit stabilizer-dependent changes in phase assemblage and microstructure that influence optical behavior, while shade-matched CAD/CAM systems can still yield small inter-material color differences when thickness and processing are controlled. In particular, reports evaluating multilayer pre-colored zirconias have highlighted that color accuracy and shade matching depend strongly on product line, measurement geometry, and specimen parameters, and that the optical performance of higher-yttria zirconias is strongly affected by their phase constitution and microstructure. In this context, the present study adds value by combining layer-resolved colorimetric outcomes (CIELAB and ΔE_00_) with corroborating compositional (EDS), crystallographic (XRD), and microstructural (FE-SEM/line-intercept grain size) evidence within the same commercial family, thereby providing a more integrated rationale for why the grades can differ substantially in stabilizer content and microstructure yet remain visually compatible in a matched shade [6,7,23,31,32].

Recent methodological work has also emphasized that translucency complicates instrumental color measurement because background show-through and device/geometry differences can meaningfully shift measured CIELAB values. Akl et al. validated clinical color measuring instruments for dental research, and Tango et al. proposed strategies to harmonize color measurements using translucent tooth-colored materials. This supports our standardized protocol; however, it also indicates that absolute CIELAB values and ΔE_00_ should be interpreted in the context of instrument geometry and background, particularly for high-translucency zirconia grades [24,30].

Furthermore, recent studies on multilayer pre-colored zirconia have shown that shade accuracy is thickness-dependent and can vary with the CAD/CAM position within the multilayer blank (nesting), which changes the relative contribution of each layer to the restoration. Kang et al. reported thickness effects on color accuracy in high-translucency multilayer zirconias, and Pongtongkham et al. demonstrated that both thickness and CAM position in Ultra-Translucent Multilayer zirconia influence color aspects. By standardizing specimen thickness (1.50 mm) and performing layer-matched third-to-third comparisons, we reduced these sources of variability, which likely contributed to the low inter-grade ΔE_00_ values observed. Nevertheless, these reports highlight the need to confirm cross-grade compatibility in anatomically shaped restorations with non-uniform thickness and clinically relevant nesting [33,34,35,36].

From a materials-engineering viewpoint, recent reviews and experimental work on graded zirconia systems provide a useful framework for interpreting why stabilizer-driven differences in phase constitution and microstructure do not necessarily translate into perceptible shade differences. Cesar et al. reviewed recent advances in dental zirconia and emphasized the coupled effects of yttria content, processing, phase composition, and grain size on optical and mechanical behavior. Inokoshi et al. characterized layer characteristics in strength-gradient multilayered yttria-stabilized zirconia, and Maharishi et al. reported light transmission and composition in color- and strength-graded zirconia; more recently, Vardhaman et al. described optical and mechanical properties across multi-transition zones in translucent zirconia. Taken together, these studies are consistent with the present findings: while the 3Y/4Y/5Y grades display clear stabilizer and microstructure-dependent differences, the manufacturer’s multilayer shading strategy can maintain layer-matched color alignment across grades under controlled reflectance conditions [31,32,37,38].

Several limitations must be considered when translating these findings to clinical situations. First, color measurements were performed on flat, polished discs of uniform thickness (1.50 mm) on a neutral gray background, which isolates intrinsic behavior but does not replicate anatomical curvature, thickness gradients, or the optical influence of underlying substrates and luting agents. Because zirconia color appearance is thickness- and background-dependent, shade compatibility observed on gray may not fully predict outcomes over black and white backgrounds (used to evaluate background/contrast effects) or over clinically relevant dark substrates (e.g., the oral cavity, discolored dentin, and metal cores) and after cementation, particularly for high-translucency grades where background show-through can be more pronounced. Second, aging protocols were not included; hydrothermal and/or thermomechanical aging may alter microstructure-related optical behavior and surface characteristics depending on zirconia composition and processing, potentially affecting long-term appearance. Third, measurements relied on one instrument and geometry; standardized, inter-device variability and the interaction between instrument geometry and specimen translucency are recognized challenges in dental colorimetry. Future studies should therefore examine the following: (1) cemented restorations on dentin-like substrates across clinically relevant cement shades and thicknesses, including measurements on black/white backgrounds; (2) crown-shaped specimens with realistic curvatures and variable thickness; and (3) ΔE_00_ stability following artificial aging and different finishing strategies (polished vs. glazed vs. stained), ideally complemented by controlled visual assessments to connect instrumental thresholds with clinical perception [3,5,7,28,29,30,33,34,35,36].

Considering the above limitations, the results support two clinically useful conclusions. First, the Katana™ multilayer system produces a consistent and clearly perceptible incisal-to-cervical gradient within each grade (UTML, STML, and HTML), reflecting an effective built-in polychromatic design. Second, when compared at equivalent regions under standardized thickness and finishing, UTML, STML, and HTML remain layer-matched in color (ΔE_00_ < PT), indicating that clinicians can reasonably combine grades within extended rehabilitations without introducing visible shade seams, while still selecting material grade primarily according to mechanical indication and clinical risk profile described in zirconia guidelines. The integration of EDS–XRD–grain size evidence further supports that the observed color compatibility is maintained despite stabilizer-driven differences in phase constitution and microstructure [4,19,37,38].

## 5. Conclusions

The present findings support the following conclusions:

1. All three multilayer zirconia grades (UTML, STML, and HTML; shade A2) exhibited a consistent incisal-to-cervical CIELAB gradient, characterized by a pronounced decrease in lightness (L*) and progressive increases in redness (a*) and yellowness (b*) from the upper (incisal) to the lower (cervical) thirds.
2. The built-in multilayer architecture generated a pronounced intra-material color gradient between layers (incisal–middle ΔE00 = 4.16–4.23; incisal–cervical ΔE_00_ = 5.68–5.96; and middle–cervical ΔE_00_ = 1.95–2.09), confirming an optically effective polychromatic transition within each zirconia grade.
3. Layer-matched cross-grade color compatibility was high. The inter-material ΔE00 values between UTML, STML, and HTML in the same region ranged from 0.20 to 0.72 and remained below the commonly used perceptibility threshold (ΔE_00_ ≈ 0.8). These results suggest that different yttria grades within this multilayer system can be combined in large rehabilitations without visible shade seams when corresponding layers are aligned and clinically comparable thickness and surface finishing are maintained.
4. Compositional and microstructural characterization confirmed the expected stabilizer-dependent hierarchy without compromising shade compatibility. EDS showed a stepwise reduction in Y_2_O_3_ content (UTML: 12.21 ± 0.09 wt%; STML: 10.04 ± 0.13 wt%; HTML: 7.07 ± 0.11 wt%). XRD demonstrated a corresponding shift from higher cubic contributions (UTML) toward predominantly tetragonal patterns (HTML) without detectable monoclinic zirconia, and grain size followed the same trend (UTML: 3.46 ± 0.85 µm; STML: 2.08 ± 0.33 µm; HTML: 0.77 ± 0.07 µm).

## Figures and Tables

**Table 1:** Manufacturer-declared yttria content, flexural strength, and recommended clinical indications for the three multilayered zirconia materials evaluated in this study.

Zirconia Material	Yttria Content (mol%)	Flexural Strength (MPa)	Translucency/Indication
UTML (Ultra-Translucent Multilayered)	~5 mol% Y^2^O^3^ (5Y)	~600–800 MPa	Highest translucency: indicated for anterior veneers/inlays and esthetic single crowns.
STML (Super-Translucent Multilayered)	~4 mol% Y^2^O^3^ (4Y)	~800–1000 MPa	High translucency: indicated for single crowns and short-span bridges (anterior or posterior).
HTML (High-Translucent Multilayered)	~3 mol% Y^2^O^3^ (3Y)	~1000–1200 MPa	Moderate translucency (more opaque): indicated for long-span or high-stress bridges.

**Table 2:** Manufacturer-recommended sintering parameters for the three multilayered zirconia grades.

Zirconia Grade	Peak Temperature (°C)	Holding Time at Peak (min)	Total Cycle Time * (h)
UTML (5Y)	1500	120	7 h 40 min
STML (4Y)	1550	120	7 h 45 min
HTML (3Y)	1550	120	7 h 45 min

* Total cycle time includes heating at 10 °C min^−1^, dwell, and natural cooling to <200 °C before removal. All firings were performed in the same high-temperature furnace (TT-1800; Nabertherm GmbH, Lilienthal, Germany) calibrated to ±2 °C across the working chamber.

**Table 3:** Mean (±SD) CIELAB coordinates (L*, a*, b*) of multilayer zirconia materials by layer *.

Coordinate	Layer	Zirconia (UTML) Mean ± SD	Zirconia (STML) Mean ± SD	Zirconia (HTML) Mean ± SD
L*	Upper	97.81 ± 2.90	96.84 ± 1.96	96.59 ± 1.81
L*	Middle	91.61 ± 0.89	90.74 ± 0.16	90.50 ± 0.13
L*	Lower	90.38 ± 0.51	90.10 ± 0.18	89.88 ± 0.16
a*	Upper	1.00 ± 0.02	0.90 ± 0.01	1.00 ± 0.01
a*	Middle	1.50 ± 0.03	1.40 ± 0.01	1.40 ± 0.01
a*	Lower	1.90 ± 0.03	1.80 ± 0.01	1.90 ± 0.01
b*	Upper	26.93 ± 0.36	26.77 ± 0.16	27.04 ± 0.14
b*	Middle	31.52 ± 0.46	31.11 ± 0.13	31.79 ± 0.11
b*	Lower	36.38 ± 0.54	36.24 ± 0.13	36.59 ± 0.12

* Values represent means and standard deviations (n = 12 per material and layer) of the CIELAB coordinates for three multilayer zirconia systems: Ultra-Translucent Multilayer (UTML), Super-Translucent Multilayer (STML), and High-Translucent Multilayer (HTML) zirconia.

**Table 4:** CIEDE2000 color differences (ΔE_00_) computed from mean CIELAB coordinates (Equation (1); KL = KC = KH = 1).

(a) Intra-Material ΔE_00_ (Between Layers, Within Each Zirconia Grade)			
Layer Comparison	UTML	STML	HTML
Incisal–Middle	4.23	4.16	4.23
Incisal–Cervical	5.96	5.68	5.68
Middle–Cervical	2.08	2.09	1.95
**(b) Inter-Material ΔE_00_ (Between Zirconia Grades, Within the Same Layer)**			
**Material Pair**	**Incisal**	**Middle**	**Cervical**
UTML–STML	0.58	0.57	0.2
UTML–HTML	0.72	0.7	0.32
STML–HTML	0.21	0.32	0.2

**Table 5:** EDS-derived oxide composition (wt%) of multilayer zirconia grades (UTML, STML, and HTML).

wt%	UTML	STML	HTML
Y_2_O_3_	12.21 ± 0.09	10.04 ± 0.13	7.07 ± 0.11
ZrO_2_	87.79 ± 0.09	89.96 ± 0.13	92.93 ± 0.11

## Data Availability

The original contributions presented in this study are included in the article. Further inquiries can be directed to the corresponding author.
